# Multimodal Physiotherapy Interventions for Cervical Myofascial Pain (2019-2025): A Structured Narrative Literature Review of Randomized Trials

**DOI:** 10.7759/cureus.96752

**Published:** 2025-11-13

**Authors:** Katerina Simati, Anteia Paraskeva, Giorgos Tzigkounakis

**Affiliations:** 1 Department of Research, Health and Resilience Institute, Athens, GRC; 2 Department of Anesthesia and Pain Medicine, Medical School, National and Kapodistrian University of Athens, Athens, GRC; 3 Departments of Anesthesia and Pain Medicine, Aretaieion University Hospital, National and Kapodistrian University of Athens, Athens, GRC

**Keywords:** cervical myofascial pain syndrome, dry needling, exercise therapy, extracorporeal shockwave therapy, manual therapy, myofascial trigger point (mtrp), pain management, physiotherapy, trigger point

## Abstract

Cervical myofascial pain syndrome (MPS) causes substantial pain and disability, but recent physiotherapy evidence remains fragmented. This structured narrative literature review synthesized randomized controlled trials (RCTs) from 2019 to 2025 on non-pharmacological physiotherapy interventions for cervical myofascial pain. PubMed and PEDro were searched for English-language RCTs, and reporting followed the Scale for the Assessment of Narrative Review Articles (SANRA). Sixty-seven trials were included.

Dry needling (DN) was most studied, with consistent short- to mid-term improvements in pain, pressure pain threshold (PPT), and range of motion (ROM), especially when paired with exercise or manual therapy. Myofascial release methods (ischemic compression, cupping, instrument-assisted soft tissue mobilization, integrated neuromuscular inhibition technique) yielded short-term analgesia and functional gains, with some protocols showing better retention. Structured exercise produced the most reliable benefits and enhanced outcomes when combined with DN or release techniques. Extracorporeal shockwave therapy (ESWT) and transfer of energy capacitive and resistive therapy (TECAR) performed comparably to DN in several trials, though long-term efficacy and cost-effectiveness data are limited. Laser, transcutaneous electrical nerve stimulation (TENS), and microcurrent showed mixed but generally positive effects. Multimodal programs outperformed single-modality care. Future research should adopt standardized, sham-controlled designs with extended follow-up periods to establish long-term efficacy and cost-effectiveness.

## Introduction and background

Neck pain is a significant global health challenge, affecting over 200 million individuals in 2020 and projected to increase by one-third by 2050 [1]. The prevalence is higher in women and peaks between the ages of 45 and 74. Within this burden, myofascial pain syndrome (MPS) is a frequent contributor to non-specific neck pain. Multiple studies across populations and settings show a high prevalence of cervical myofascial trigger points (MTrPs), especially in the trapezius and levator scapulae [2-4].

MPS, one of the most common causes of musculoskeletal pain [5], is characterized by the presence of MTrPs, which produce local or referred pain, restricted range of motion (ROM), and functional limitation [6]. The condition is usually attributed to overuse, trauma to the neck musculature, postural mechanics, and ergonomic stressors [7], while nutritional deficiencies, psychological stress, behavioral patterns, and lifestyle factors have also been characterized as risk factors [8,9]. Many of these factors interact synergistically, underscoring the need for a multidimensional therapeutic approach [10,11].

Diagnosis relies primarily on subjective clinical findings, including palpation of taut bands, reproduction of the patient’s referral pattern, and often elicitation of a local twitch response (LTR), although criteria are not fully standardized or consistently accepted [12-15]. To support diagnosis and evaluate outcomes, standardized tools are widely applied in both research and practice, most commonly the Visual Analogue Scale (VAS), the Neck Disability Index (NDI), and pressure algometry for pain threshold (PPT).

Instrumental methods such as surface electromyography (sEMG), ultrasound elastography [16-20], and magnetic resonance elastography [21,22] have been explored, though their validity remains inconsistent [23]. In vivo microdialysis indicates that active MTrPs present a distinct biochemical milieu with reduced pH and elevated inflammatory mediators, neuropeptides, and catecholamines [24]. These findings align with the hypothesis of an ischemic and hypoxic microenvironment within muscle tissue [25] and are consistent with Simons' integrated trigger point hypothesis, which links contractile, metabolic, and biochemical cascades. Complementary models include the Cinderella hypothesis of type I fiber overload during prolonged low-level exertions and neuromuscular junction dysfunction with excessive acetylcholine release [8]. More recent perspectives emphasize central sensitization, neurogenic inflammation, and fascia densification, suggesting that MPS reflects an interplay between peripheral and central mechanisms rather than a single pathway [8].

Management of MPS aims to relieve pain and address contributing factors. Pharmacological options commonly include nonsteroidal anti-inflammatory drugs (NSAIDs), muscle relaxants, and, in selected cases, benzodiazepines or antidepressants, although their effectiveness is supported by varying levels of evidence [26,27]. Other approaches include topical lidocaine patches and injection therapies such as corticosteroids or botulinum toxin. Among these, trigger-point injections are regarded as one of the most effective methods for inactivating MTrPs and pain management, although they may be associated with adverse effects such as muscle weakness, skin atrophy, nerve injury, dysphagia, and respiratory compromise [28-31].

In physiotherapy, standard care commonly includes assessment, therapeutic exercise, manual techniques, patient education, functional training, massage, and basic physical agents such as heat, cold, electrotherapy, and ultrasound [32-34]. Its exact content varies across countries and settings, and it is considered the clinical baseline for comparison. In this review, the term physiotherapy interventions refers broadly to structured therapeutic approaches delivered by physiotherapists, whereas physiotherapy modalities denote specific physical or biophysical methods applied within those interventions. The term "advanced physiotherapy modalities" describes techniques that extend beyond standard care in many systems, including dry needling (DN), extracorporeal shockwave therapy (ESWT), transfer of energy capacitive and resistive therapy (TECAR), selected forms of photobiomodulation such as high-intensity laser therapy, and specialized manual or exercise-based techniques. The classification is used flexibly, as what is considered advanced may vary by context and country.

This structured narrative review synthesizes randomized controlled trials (RCTs) (2019-2025) on physiotherapy interventions for cervical MPS, with particular emphasis on these advanced modalities, in order to map current evidence, compare outcome trends across intervention classes, and inform clinical decision-making.

## Review

Methodology

This structured narrative literature review was conducted in accordance with the Scale for the Assessment of Narrative Review Articles (SANRA) quality criteria. A formal risk-of-bias assessment was not performed because this review aimed to provide an integrative synthesis rather than a quantitative evaluation. The review focused on non-pharmacological physiotherapy interventions for adults with cervical MPS associated with trigger points, with particular emphasis on physiotherapy modalities often considered beyond standard care, including DN, ESWT, TECAR, manual therapy, and photobiomodulation using high- and low-level laser therapies.

**Figure 1:**
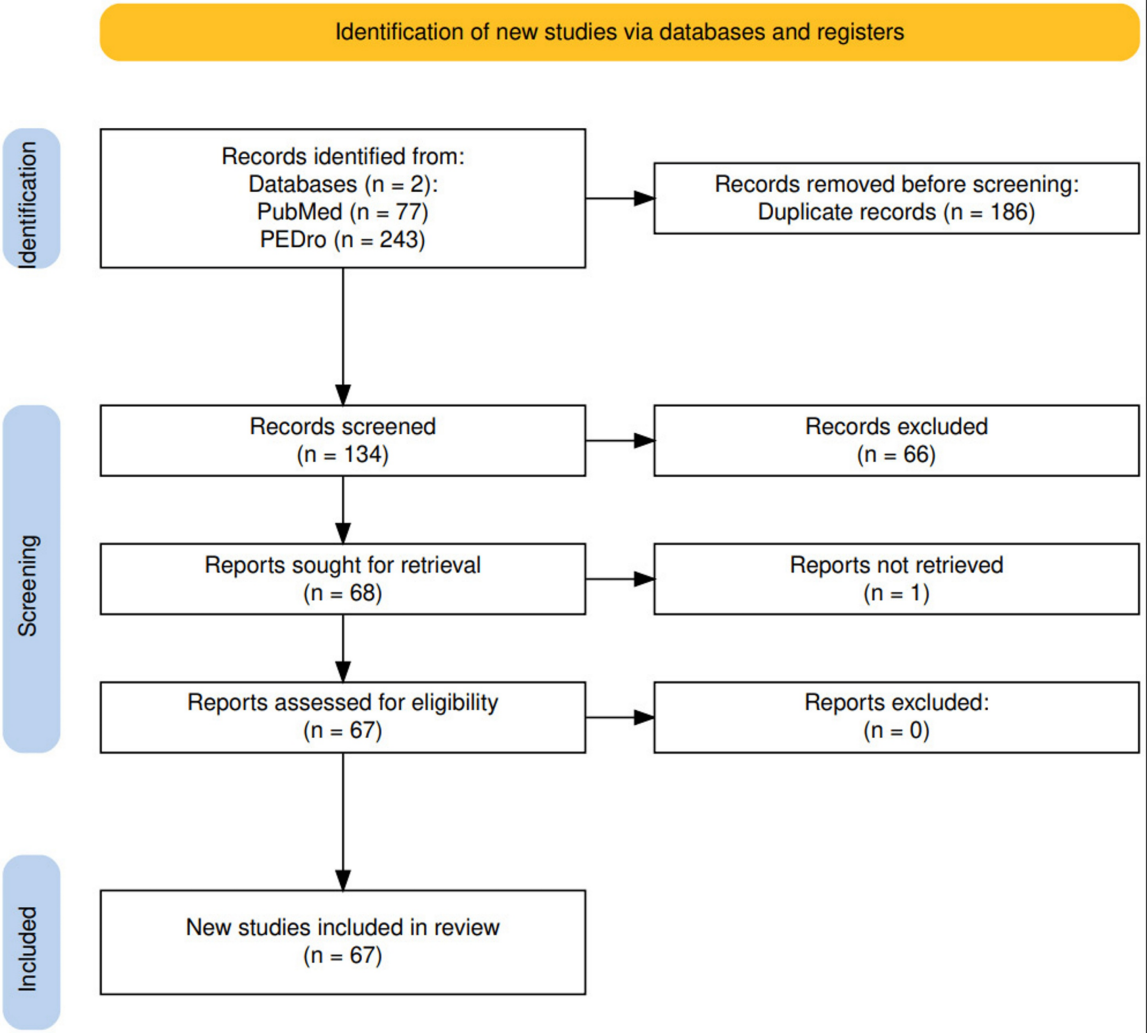
Flow diagram of the study selection

A comprehensive search was conducted across PubMed and PEDro databases. The last search was conducted on July 20, 2025. The search was restricted to RCTs published in English between 2019 and 2025. Search terms combined keywords related to the anatomical region, the pathology of myofascial pain and trigger points, the interventions of interest, and the study design (Table 1). Boolean operators (“AND,” “OR”) were applied to structure the queries. To increase specificity, studies addressing unrelated conditions such as pelvic pain, headache, migraine, or pelvic disorders were excluded through keyword filtering. Reference chaining from key publications and cross-referencing among relevant clinical studies were applied to broaden coverage. Grey literature and non-peer-reviewed sources were excluded to maintain methodological rigor. A flow diagram was used to illustrate the study selection process (Figure 1), and the full list of included RCTs is presented in Table 2.

**Table 1:** Search strategy

Keywords
"neck pain", "cervical pain", "upper trapezius", "trigger point", "trigger points", "myofascial trigger point", "MTrP", "TrP", "TrPs", "myofascial syndrome", "myofascial syndromes", "myofascial pain", "manual therapy", "myofascial release", "IASTM", "Instrument Assisted Soft Tissue Mobilization", "instrument assisted", "soft tissue", "TECAR therapy", "capacitive-resistive electric transfer", "capacitive-resistive monopolar radiofrequency", "radiofrequency diathermy", "shockwave therapy", "extracorporeal shockwave therapy", "dry needling", "massage", "ischemic compression", "ultrasound", "diathermy", "thermotherapy", "heat therapy", "TENS", "transcutaneous electrical nerve stimulation", "laser therapy", "exercise", "magnetotherapy", "physical therapy modalities", "randomized controlled trial", "RCT"

Results

A total of 67 RCTs were included, of which 31 specifically examined DN for cervical myofascial pain [35-65]. Most trials reported significant improvements in pain, PPT, and ROM, particularly when targeting active MTrPs with deep DN [38,42,44,46,49,51,57,60]. Imaging and EMG studies further confirmed reductions in muscle stiffness, trigger point diameter, and abnormal electrical activity in the upper trapezius [51,53,60]. Combining DN with therapeutic exercise or myofascial release consistently enhanced outcomes [35,50,53].

However, approximately one-third of studies found DN to be more effective than other modalities such as exercise, ultrasound, ESWT, or kinesiotaping [37,39,41,43,45,54,61,62,64-66]. Electroacupuncture, also described as DN with electrical stimulation or intramuscular electrical stimulation, consistently produced short-term improvements in pain and function [36]. While one study reported faster clinical gains with this approach, no sustained advantage over conventional DN was observed [37]. Collectively, DN trials indicate consistent short-term improvements in pain and mobility, particularly when targeting active trigger points, though durability beyond the medium term remains uncertain.

Myofascial release techniques, including ischemic compression and cupping, consistently produced short-term improvements in pain, PPT, ROM, and quality of life, while also reducing muscle stiffness [35,40,48,55,56,67,68]. Position release therapy provided slightly longer-lasting effects, up to four weeks, and improved tissue elasticity and muscle thickness [69]. Instrument-assisted soft tissue mobilization (IASTM) showed comparable or superior efficacy to manual release, particularly for pain reduction, and, when combined with conventional physiotherapy, further enhanced PPT, ROM, and patient satisfaction [70,71]. Overall, release-based methods produce consistent short-term benefits, with some approaches (e.g., positional release, IASTM) showing modestly longer-lasting or superior effects.

Structured exercise improved pain, function, and mobility in patients with MTrPs or cervical MPS [50,53,72-74]. When combined with techniques such as the integrated neuromuscular inhibition technique (INIT) or DN, exercise further enhanced analgesia and reduced trapezius trigger point thickness or diameter [53,72]. Exercise was the main contributor to long-term gains in pain, ROM, and function, even when paired with other therapies [53,72]. Cervical mobilization, neurodynamic techniques, and INIT also reduced pain and improved PPT, ROM, NDI, and quality of life [72,75-80]. INIT outperformed spray-and-stretch [76], while combining myofascial release or ischemic compression with neurodynamic or re-education techniques enhanced analgesic and functional outcomes [75,78,79]. Collectively, exercise-based interventions demonstrated consistent advantages across pain, mobility, and function, both alone and when integrated with manual or instrumental techniques.

Studies on ESWT reported reductions in pain and improvements in PPT, ROM, and NDI, often within 3-7 days [71,81,82]. ESWT produced results comparable to DN [41,45], outperformed sham treatment [83], and in one trial was superior to conventional physiotherapy and kinesiotaping [81].

Radiofrequency (TECAR) therapy produced pain relief after the first session and improved function and ROM [84]. Some studies suggested it was superior to therapeutic ultrasound in prolonging benefits and improving NDI [85], whereas others reported no significant difference compared to exercise [86]. Therapeutic ultrasound also reduced pain and improved ROM, with greater effects in high-power protocols [67,87]. In one study, ultrasound phonophoresis demonstrated results comparable to DN [40], while another found that both therapeutic ultrasound and TECAR therapy were effective for latent MTrPs, with TECAR demonstrating a longer duration of action [85].

Electrotherapy interventions reduced pain, increased PPT, and improved ROM and function [88-91]. TENS combined with myofascial release was more effective than either alone [89], and AL-TENS produced greater ROM gains than C-TENS [88]. Microcurrent therapy (MCT) and interferential currents (IFC), particularly at 4 Hz, provided stronger analgesia and better sleep quality than controls [90,91]. Overall, electrical stimulation generally improves pain, PPT, ROM, and function, yet effects are protocol-dependent, with AL-TENS and low-frequency IFC often outperforming conventional TENS.

Laser therapies, also known as photobiomodulation therapy, showed mixed results. In a small study, high-intensity laser therapy (HILT) was comparable to DN [65]. Other studies demonstrated that low-level laser therapy (LLLT) was superior to placebo for pain and PPT [92,93], while Nd:YAG laser with pressure techniques outperformed placebo [94]. However, one study found no added benefit when the laser was combined with standard therapy [95]. Moreover, a study found that although photobiomodulation combined with DN did not improve pain outcomes beyond DN alone, it reduced muscle activation, whereas unexpectedly, DN outside trigger points yielded the greatest improvements in pain and disability [52].

The spray-and-stretch technique reduced pain and improved PPT, ROM, and function. Its analgesic effects were comparable to DN and INIT, though INIT achieved greater gains in PPT and disability [54,76]. Compared with sustained pressure, spray-and-stretch showed better improvements in ROM, disability, and psychological outcomes, while sustained pressure provided stronger pain relief [96]. Kinesiotaping was also effective for reducing pain and improving ROM and function, with outcomes similar to DN [39,61,64,97]. Some studies, however, found no added benefit when combined with exercise [73,98]. A multicenter RCT reported that EDF kinesiotaping was superior to sham in short-term reductions in pain, trigger points, and disability [99].

Across modalities, structured exercise consistently enhanced outcomes when used as a standalone approach or combined with manual or instrumental therapies.

**Table 2:** Summary of included randomized controlled trials S-b: single-blind, D-b: double-blind, Tr-b: triple-blind, SWE: shear wave elastography, INIT: integrated neuromuscular inhibition technique, P-IRT: post-isometric relaxation technique, CMT: cervical mobilization technique, TE: therapeutic exercise, MR: myofascial release, PR: pressure release, PB: phonophoresis of betamethasone, RF: radiofrequency, LTR: local twitch response, N-LTR: non-local twitch response, SG: study group, CG: control group, CT: conservative treatment, PRT: positional release therapy, TM: therapeutic massage, DNP: dry needling & photobiomodulation, PBM: photobiomodulation,  ESWT: extracorporeal shock wave therapy, KT: kinesiotaping, CTTM: court-type traditional Thai massage, THE: Thai hermit exercise, ThM: Thai massage, ME: muscle energy, HF-PENS: high-frequency percutaneous electrical nerve stimulation, LF-PENS: low-frequency percutaneous electrical nerve stimulation, MTrPs: myofascial trigger points, LTrPs: latent trigger points, EDF: epidermis-dermis-fascia kinesiotaping, KSCT: kinesiotaping with the space correction technique, KMIT: kinesiotaping with the muscle inhibition technique, IMES: intramuscular electrical stimulation, SDN: superficial dry needling, DDN: deep dry needling, EG: exercise group, TENS: transcutaneous electrical nerve stimulation, AL-TENS: acupuncture-like TENS, C-TENS: conventional TENS, S-TENS: sham TENS, CTrPs: control trigger points, DTFM: deep transverse friction massage, WBV: whole-body vibration, HPPT-US: high-power pain-threshold ultrasound, PT: physiotherapy, DTM: deep tissue massage, HILT: high-intensity laser therapy, US: ultrasound, DCD: digital capacitive diathermy, CRG: capacitive-resistive diathermy therapy group, PG: placebo group, IASTM: instrument-assisted soft tissue mobilization, CS-S: cold-spray stretching, BC: blood circulation, SP: sonographic parameters, IFC: interferential current, PPRT: progressive pressure release technique, ANDI: Arabic Neck Disability Index.

	Studies	Design	Patients (n)	Evaluation method	Interventions	Duration	Follow-up	Results (improvements)	p-value
1.	Ziaeifar et al. (2019) [56]	RCT	33	VAS, NPQ, DASH	2 groups: DN vs TPC	3 sessions (1 w)	2 w, 3 mo	Yes, in all parameters, VAS (DN > TPC)	p < 0.0001; VAS (DN > TPC): p = 0.02
2.	Tabatabaiee et al. (2019) [40]	RCT	60	VAS, PPT, ROM	3 groups: DN, PR, PB	PR & PB 3×/w; DN 2×/w; all for 2 w	24 h	Yes, DN & PB had better effects than PR	VAS (DN & PB): p < 0.001; ↑PPT (DN & PB): p < 0.001; ROM (DN & PB): p < 0.001
3.	Alguacil-Diego et al. (2019)[84]	D-b. RCT	24	VAS, NDI, ROM	2 groups: RF vs RF-placebo	8 sessions 2×/w	No	Yes, pain reduction in RF group vs placebo	VAS (RF): p < 0.001; NDI: p < 0.05; right rotation (RF): p = 0.026
4.	Hakim et al. (2019) [38]	RCT	26	VAS, PPT, ROM, NDI	2 groups: DN LTR vs DN N-LTR	3 sessions (1 w)	4 w	Yes, in VAS, PPT, ROM: N-LTR > LTR	VAS: p = 0.03; PPT: p = 0.049; ROM: p < 0.05; NDI: p = 0.935
5.	Manafnezhad et al. (2019) [45]	S-b. RCT	70	NPRS, PPT, NDI	2 groups: DN, ESWT	3 sessions 1×/w	1 w	Yes, equally effective	NPRS: p < 0.001; PPT: p < 0.001; NDI: p < 0.001
6.	Doğan et. al. (2019) [39]	S-b. RCT	42	VAS, PPT	2 groups: DN vs KT	3 sessions 1-2×/w	5 d, 4 w	Yes, equally effective	VAS: p < 0.001; PPT (DN): p = 0.006; (KT): p = 0.007
7.	Luan et al. (2019) [41]	S-b. RCT	65	VAS, PPT, NDI, SWE	2 groups: DN vs ESWT	3 sessions 1×/w	1 mo, 3 mo	Yes, equally effective	VAS: p < 0.01; PPT: p < 0.01; NDI: p < 0.01; SWE: p < 0.01
8.	Waseem et al. (2020) [92]	RCT	54	NPRS, ROM	2 groups: LLLT + CT vs CT	3 sessions 3×/w	2 w, 4 w	Yes, in both groups, LLLT + CT had greater pain improvement	p < 0.05; NPRS (LLLT + CT > CT): p < 0.05
9.	Alayat et al. (2020) [94]	RCT	50	VAS, PPT, ROM	2 groups: laser + PPRT, placebo laser + PPRT	12 sessions 3×/w	No	Yes, in both groups, but laser + PPRT group had much greater improvements	p < 0.05
10.	Nasb et al. (2020) [68]	S-b. RCT	24	PPT, NDI, ROM	3 groups: cupping, IC, (IC + C)	8 sessions 2×/w	4 w	Yes, the IC + C group was superior in PPT & NDI	PPT: p < 0.05; NDI: p < 0.01; ROM: p < 0.05
11.	Lytras et al. (2020) [72]	S-b. RCT	40	VAS, NDI, PPT, ROM, SF-36	2 groups: TE + INIT vs TE	40 sessions 4×/w (10 w)	6 mo	Yes, but the TE + INIT group was superior	VAS: p < 0.001; NDI: p < 0.001; PPT: p < 0.05; SF-36: p < 0.05
12.	Alghadir et al. (2020) [79]	S-b. RCT	60	VAS, PPT	3 groups: MET + IC, MET + CT, CT	1 session	2 w	Yes, in all groups, but the MET + IC group was superior	p < 0.05
13.	Zardoust et al. (2021) [100]	S-b. RCT	55	VAS, PPT, SF-36	3 groups: KT + exercise, sham KT + exercise	4 sessions (20 d)	1 mo	Yes, but no statistically important differences between groups	p = 0.001
14.	Stieven et al. (2021) [55]	S-b. RCT	44	NPRS, PPT	3 groups: DN, MR, sham DN	1 session	10’ after	Yes, substantial changes only in groups DN, MR	↑ PPT (DN, MR): p = 0.001; ↓NPRS (DN, MR): p < 0.001; sham DN: p = 0.008
15.	Anwar et al. (2021) [78]	RCT	50	NPRS, ROM	2 groups: IC vs IC + NMR	8 sessions 4×/w	No	Yes, but the IC + NMR group was superior in NPRS and ROM	p < 0.001; NPRS/ROM (IC + NMR > IC): p < 0.001
16.	Yasar et al. (2021) [61]	RCT	88	VAS, NDI, PPT, GPE	3 groups: CG, KT, DN	4 sessions 2×/w	No	Yes, substantial changes only in DN, KT groups	p < 0.05; p > 0.05 (between groups)
17.	Choksi et al. (2021) [101]	RCT	66	VAS, ROM (side flexion)	3 groups: DTFM, IC, CT	3 sessions	No	Yes, equally effective in pain and ROM in 3 groups	p = 0.0001
18.	Emshi et al. (2021) [59]	S-b. RCT	81	NPRS, PPT, ROM	3 groups: DN, IASTM, CG	4 sessions 2×/w	1 mo	Yes, DN & IASTM in VAS/PPT. IASTM was superior in ROM	NPRS (DN/IASTM): p < 0.05; PPT (DN/IASTM): p < 0.05 ROM (IASTM>DN): p < 0.05
19.	Bethers et al. (2021) [69]	RCT	60	VAS, PPT, ultrasound, SWE	2 groups: TM vs PRT	1 session	48 h	Yes, for both, PRT > MT in improvement retention	VAS: p < 0.0001; PRT: p < 0.0001; (PRT > MT) U + SWE: p < 0.0001
20.	Ebadi et al. (2021) [88]	RCT	60	VAS, DASH, PPT, ROM	3 groups: AL-TENS vs C-TENS vs S-TENS	5 continuous sessions	3 mo	Yes, in VAS, PPT DASH (AL-TENS, C-TENS > Sham). AL-TENS > C-TENS, Sham: ↑ ROM, faster & consistent results	p < 0.05
21.	Battecha et al. (2021) [90]	RCT	28	Pain, ROM, NDI, PPT	2 groups: MCT + CT vs CT	6 sessions 2×/w	No	Yes, in both groups, but MCT was superior	p < 0.05
22.	Dalpiaz et al. (2021) [52]	S-b. RCT	43	VAS, PPT, NDI, RMS values	3 groups: DNP: (DN + PBM on DN); (DN + PBM off DNout); (DN outside TrP + PBM off)	1 session	10’ and 30’ after, 1 w, 1 mo	Yes, similar effects in pain for DNP, DN, DN-out, but DNP had better results in RMS	VAS (DNP & DN-out): p < 0.001; NDI: p < 0.001; PPT: p = 0.139; RMS (DNP): p < 0.001
23.	Gattie et al. (2020) [43]	D-b. RCT	77	NDI, VAS	2 groups: DN vs sham DN	6 sessions 2×/w	4 w, 6 mo, 12 mo	Yes, but no difference adding DN	NDI: p = 0.69; VAS: p = 0.37; p = 0.10 (24 h)
24.	Boonruab et al. (2021) [74]	S-b. RCT	46	VAS, ROM	2 groups: CTTM vs THE	6 sessions 3×/w	7 d, 11 d	Yes, CTTM > THE in VAS, CTTM = THE in ROM	VAS: p < 0.05; CTTM > THE ROM: p < 0.05
25.	Buttagat et al. (2021) [102]	S-b. RCT	45	VAS, PPT, NDI, ROM	3 groups: TM, ME, CG	8 sessions 4×/w	1 d	Yes, equally effective	VAS/PPT/NDI/ROM (TM, ME): p < 0.05
26.	Hernandez et al. (2021) [36]	S-b. RCT	40	VAS, PPT	2 groups: LF-PENS, HF-PENS	2 sessions 1×/w	1 mo	Yes, similar results in both groups (VAS)	VAS: p < 0.01; PPT: p = 0.241
27.	Sánchez-Infante et al. (2021) [44]	D-b. RCT	51	PPT, SWE	2 groups: DN vs sham DN (LTrPs)	1 session	After 30’, 24 h, 72 h	Yes, only in DN group	PPT (DN): p < 0.05; SWE (DN): p < 0.01
28.	Akpinar et al. (2021) [98]	RCT	71	NPRS-11, NDI, SF-36	3 groups: KSCT, KMIT, CG	4 sessions 2×/w	1 mo	Yes, KT techniques equally effective	NPRS-11: p < 0.05 (KT > CG); NDI (KT): p = 0.011; SF-36 (KT): p < 0.05
29.	Brennan et al. (2021) [37]	S-b. RCT	45	NDI, NPRS	2 groups: DN vs DN/IMES (10 Hz)	7 sessions (in 6 w)	6 w	Yes, effects persisted for 6 w	NDI (DN): p = 0.01; NDI (IMES): p < 0.001; NPRS: p = 0.02
30.	Sánchez-Infante et al. (2021) [60]	D-b. RCT	50	PPP, DS, MT	2 groups: DN vs sham DN (LTrPs)	1 session	After 30’, 24 h, 72 h	Yes, in DN group	PPP (DN): p < 0.01; DS (DN): p = 0.04; MT (DN): p = 0.04
31.	Navarro et al. (2022) [42]	D-b. RCT	180	PPT, ROM	3 groups: SDN, DDN, CG	1 session	24 h, 72 h, 7 d	Yes, for ipsilateral rotation and PPT, only in SDN & DDN groups	SDN/DDN; PPT: p < 0.001 (7 d) ROM; ↑ ipsilateral rotation: p = 0.028 (7 d)
32.	Yildirim et al. (2022) [64]	RCT	30	VAS, SF-36	3 groups: CT, KT + CT, DN + CT	10 sessions 3×/w 5 sessions DN/KT	No	Yes, equally effective in all groups	
33.	Cabrera-Martos et al. (2022) [75]	S-b. RCT	40	TrPs evaluation, VAS	2 groups: MR + ND (EG) vs CG	12 sessions 3×/w (4 w)	No	Yes, in the EG group, ↓ TrPs and pain	↓TrPs: p < 0.05 (SO, LS, SC); VAS (EG): p = 0.047
34.	Haq and Riaz (2022) [63]	RCT	30	NPRS, NDI, ROM, TrPs sensitivity	2 groups: DN vs IASTM	4 sessions 2×/w	No	Yes, better results in DN group	p < 0.05 (DN > IASTM)
35.	Almushahhim et al. (2022) [62]	S-b. RCT	31	NPRS, NDI, SF-36, BDI	2 groups: DN + exercise vs exercise	6 sessions 3×/w +1 DN	No	Yes, equally effective	p ≤ 0.05
36.	Martín-Sacristán et al. (2022) [57]	D-b. RCT	65	VAS	3 groups DN: non-MTrP, active-MTrP, latent-MTrP	1 session	After 1-72 h, 1 w, 1 mo	Yes, in all groups, VAS active-MTrP < non-MTrP	p < 0.01; 1 w after (VAS active-MTrP < non-MTrP): p < 0.01
37.	Thakur et al. (2022) [80]	RCT	60	NPRS, ROM, NDI	2 groups: INIT, IASTM	INIT: 3×/w IASTM: 1×/w (2 w)	No	Yes, in both groups, but INIT was superior	p < 0.0001; p < 0.05 (INIT > IASTM)
38.	Ahmad et al. (2022) [96]	D-b. RCT	54	VAS, PPT, ROM, NDI, HADS	2 groups: spray-stretch + CT vs pressure + CT	6 sessions 3×/w (2 w)	2 w	Yes, equally effective	p < 0.05
39.	Khanittanuphong and Saesim (2022) [58]	S-b. RCT	54	VAS, PPT	2 groups: DN vs DN-retention	1 session	7 d, 14 d	Yes, equally effective	VAS: p < 0.001; PPT: p < 0.001
40.	Rodríguez-Jiménez et al. (2022) [48]	S-b. RCT	50	NPRS, PPT, muscle performance (MP)	2 groups: DN vs MR	1 session	After 5’	Yes, DN was superior in PPT & MP	NPRS: p < 0.01; PPT (DN): p < 0.05; MP (DN): p = 0.03
41.	Sánchez-Infante et al. (2022) [46]	D-b. RCT	46	sEMG, PPT	2 groups: DN vs sham DN (LTrPs)	1 session	After 30’, 24 h, 72 h	Yes, only in DN group	PPT (DN): p < 0.01; sEMG (DN): p < 0.05
42.	Korkmaz and Medin Ceylan (2022) [53]	S-b. RCT	62	VAS, diameter of TrPs, NDI	2 groups: DN + EG vs EG	DN: 1×/w (3 sessions) EG: 3×/w (3 mo)	3 mo	Yes, better effects in DN + EG group	VAS (DN + EG): p < 0.001; diameter of TrPs (DN + EG): p = 0.021
43.	Hoseininejad et al. (2023) [49]	S-b. RCT	47	VAS, NDI, sEMG	2 groups: DDN vs SDN	1 session	1 w	Yes, in both groups, DDN had better results in sEMG	VAS: p < 0.05; NDI: p < 0.05; sEMG (DDN): p < 0.05
44.	Ceylan et al. (2022) [73]	S-b. RCT	57	VAS, NDI, trigger point diameter, & trapezius thickness	2 groups: KT + EG vs EG	4 sessions: KT 2×/w + 4 w exercises	1 mo	KT + EG was superior in all parameters	KT + EG: VAS: p < 0.001; NDI: p < 0.001; trigger point diameter; trapezius thickness: p < 0.05
45.	Ghulam et al. (2023) [77]	D-b. RCT	30	VAS, NDI, PPT, ROM	2 groups: P-IRT + CMT vs P-IRT	9 sessions: 3×/w	No	Yes, in both groups, but P-IRT + CMT group was superior in pain and disability	p < 0.05; NDI & VAS (P-IRT + CMT > P-IRT): p < 0.001
46.	Iakovidis et al. (2023) [89]	S-b. RCT	80	VAS, NDI, PPT, ROM	4 groups: MR, MR + TENS, TENS, placebo	6 sessions 2×/w	1 mo	Yes, MR + TENS group more effective in pain	MR + TENS: VAS: p < 0.001; NDI: p < 0.001; PPT: p < 0.001; ROM (lateral flexion): p < 0.001; MR: NDI: p < 0.001
47.	Candeniz et al. (2023) [71]	RCT	42	VAS, NOOS, HADS, PPT, ROM, Satisfaction of the patients	3 groups: CT, CT + ESWT, CT + IASTM	3 w: 5×/w (CT) 2×/w (ESWT, IASTM)	3 d	Yes, in all groups. CT + IASTM was superior in VAS, PPT, ROM	p < 0.001
48.	Valera-Calero et al. (2024) [51]	D-b. RCT	60	SWE, PPT	2 groups: DN vs sham in 2 points (CTrP & MTrP)	1 session	10’ after	DN ↑ PPT (CTrP) vs sham; both ↑ PPT (MTrP). No SWE change	SWE: p > 0.05; PPT (DN in CTrP): p < 0.01
49.	Sadeghnia et al. (2023) [67]	D-b. RCT	66	VAS, PPT, ROM	3 groups: DTFM, HPPTUS, WBV	1 session	No	Yes, in all groups, WBV in VAS, HPPTUS + WBV in ROM	VAS: p < 0.01; PPT: p < 0.05; ROM (CLF): p = 0.00
50.	Şah et al. (2023) [81]	S-b. RCT	84	VAS, NDI, ROM	3 groups: CT, KT, ESWT	10 sessions (CT) 5×/w 4 sessions (KT, ESWT) 2×/w	1 mo	Yes, in all groups, ESWT greater improvement in all parameters (1 mo follow-up)	p < 0.05; 1-mo follow-up (ESWT > CT, KT): p < 0.05
51.	Karagül and Saime (2024) [35]	RCT	98	VAS, PPT, NDI, ROM	3 groups: DN, IS, DN + IC	DN: 1 session; IC: 2×/w (4 w); DN + IC: 1 session; DN + 2×/w (4 w)	1 mo, 3 mo	Yes, DN + IC was superior	VAS (DN + IC): p < 0.001; PPT (DN + IC): p = 0.02; ROM: p < 0.05; NDI (DN + IC): p < 0.001
52.	Külcü et al. (2024) [99]	D.b. RCT	180	VAS, number of TrPs, ROM, NPDS	3 groups: EDF kinesio vs sham kinesio	2 sessions	2-w	Yes, EDF kinesio group was superior	VAS: p < 0.05; TrPs: p < 0.001; ROM: p = 0.001; NPDS: p < 0.001
53.	Bingölbali et al. (2024) [103]	RCT	80	VAS, PPT, NPDS, ROM, SF-36	2 groups: DTM vs CG	20 sessions 5×/w DTM: + 12 sessions, 3×/w	No	Yes, DTM had greater improvements in all parameters vs CG	DTM: VAS: p < 0.05; NPDS: p < 0.05; ↑ROM: p < 0.05; SF-36: p < 0.05
54.	Ali et al. (2024) [95]	RCT	24	NPS, PPT, ROM	2 groups: LLLT vs CG	1 session	No	Yes, in both groups	p < 0.05 in both groups; p > 0.05 between groups
55.	Mahdizadeh et al. (2024) [47]	D-b. RCT	30	VAS, NDI, COP	2 groups: DN vs sham DN	3 times in a week	15 d	Yes, in VAS & NDI (DN), no in COP	VAS (DN): p = 0.000; NDI (DN): p = 0.000; NDI (sham): p = 0.001; COP: p > 0.05
56.	Kocabal and Gündüz (2024) [93]	S-b. RCT	60	VAS, PPT, ROM, NDI, BDI	2 groups: LLLT, placebo	10 sessions 5×/w	1 mo	Yes, LLLT group had greater effects in pain and left lateral flexion	p < 0.001; VAS (SG > CG): p = 0.04; ROM-LLF (SG > CG): p < 0.01); PPT (SG > CG): p < 0.01
57.	Alattar and Alzahrani (2024) [50]	S-b. RCT	30	VAS, NDI, ROM	2 groups: DN vs PT	4 sessions 2×/w	3 w, 7 w	Yes, in NDI, ROM (DN), no in VAS	↑VAS (DN): p = 0.022; ↓NDI (DN): p = 0.003; ROM (DN): p < 0.05
58.	Yassin et al. (2024) [65]	S-b. RCT	32	VAS, NDI, ROM	2 groups: DN vs HILT	5 sessions 2×/w	2 d	Yes, equally effective	VAS: p < 0.05; NDI: p < 0.05; ROM: p < 0.05
59.	Ali Ismail et al. (2024) [76]	S-b. RCT	60	VAS	3 groups: INIT, spray-stretch, stretching	12 sessions 3×/w	No	Yes, INIT had the most significant improvements	INIT & spray-and-stretch: VAS: p < 0.05
60.	Jiménez-Sánchez et al. (2024) [85]	S-b. RCT (crossover)	19	VAS, muscle stiffness, PPT, NDI, ROM	2 groups crossover: US, diathermy (DCD)	1 session (both Interventions) with 1 w washout	1 w	Yes, US had greater effects in VAS, when DCD had in NDI	VAS (US): p = 0.005; NDI (DCD): p < 0.05
61.	Dinçer et al. (2024) [86]	D-b. RCT	36	VAS, PPT, NDI, ROM, SF-36	2 groups: CRG vs PG (+ exercises in both)	10 sessions 2-3×/w	No	Yes, in both groups. ↑ extension in CRG group, but minor differences for the rest	In both groups VAS/PPT/NDI/SF-36: p < 0.05; ROMEXT (CRG): p < 0.05
62.	Agarwal et al. (2024) [70]	S-b. RCT	31	NPRS, NDI, PPT, ROM	2 groups: IASTM vs MFR	3 sessions 3×/w	No	IASTM was superior in pain. No difference in PPT, ROM, NDI	NPRS: p < 0.05
63.	Ibrahim et al. (2024) [87]	S-b. RCT	75	VAS, ANDI, PPT, ROM	3 groups: iontophoresis MgSO4 + CT, HPPT-US + CT, CT	8 sessions 2×/w	No	Yes, in all parameters in experimental groups, limited in CT group	p < 0.0001 in all parameters in experimental groups
64.	Ustun et al. (2024) [54]	S-b. RCT	60	NRS, PPT, ROM, NDI, sEMG, US	2 groups: DN group, CS-S group	3 sessions 1×/w	No	Yes, in both groups, CS-S was superior in PPT, NDI	p < 0.05 in all groups; CS-S superior in PPT: p < 0.008; NDI: p < .028
65.	Hadizadeh et al, (2025) [66]	S-b. RCT	30	VAS, PPT, NDI, ROM, BC, SP	2 groups: DN vs IMES	3 sessions 1×/w	1 mo	Yes, in both groups, IMES was superior in ROM, BC, SP	p < 0.02; IMES (ROM/BC/SP): p = 0.00
66.	Hussein et al. (2025) [91]	Tr-b. RCT	120	NRPS, ROM, NDI, ISI	4 groups: INIT, INIT + 4 Hz, INIT + 80 Hz INIT + 130 Hz	12 sessions 3×/w	3 mo	Yes, in all groups, INIT + 4 Hz group had the greatest effects	p < 0.05 (INIT + 4 Hz); NPRS: p < 0.001; ISI: p < 0.001
67.	Vasvit et al. (2025) [82]	D-b. RCT	64	Shear modulus, VAS, NDI	2 groups: fESWT vs sham-fESWT	4 sessions 1×/w	No	Yes, in all parameters in fESWT group, but sham group had improvements in VAS & NDI	p < 0.05

Discussion

This review synthesized 67 RCTs on advanced physiotherapy interventions for cervical MPS, moving beyond conventional care. The findings indicate that several modalities offer clinically meaningful benefits, but their comparative value depends on duration of effect, feasibility, and integration with exercise. To interpret the evidence, three dimensions are particularly useful, efficacy hierarchy, time course of benefits, and clinical applicability.

Dry Needling and Invasive Approaches

DN [35-65] consistently demonstrated short- and medium-term improvements in pain, PPT, and ROM [38,42,44,46,49,51,53,57,60,65]. Benefits are enhanced when DN targets active trigger points and is combined with therapeutic exercise or manual release techniques [50,53]. Systematic reviews support these findings, indicating that DN combined with physiotherapy reduces pain in both the short and medium terms [104,105]. Several studies also suggest that DN is as effective as local anesthetic injections and may provide better long-term results than corticosteroid injections for the deactivation of trigger points [106]. Nevertheless, while DN is generally low cost and widely accessible, its effectiveness may be partly contingent on practitioner skill and, in most cases, accurate trigger point localization [107,108]. Moreover, interpretation should remain cautious given variability in protocols, control conditions, and follow-up durations across DN trials. Electroacupuncture interventions, combining DN with intramuscular or percutaneous electrical stimulation, have demonstrated reliable reductions in pain and disability in myofascial pain of the upper trapezius and neck. While the addition of electrical current may facilitate earlier symptomatic relief, long-term outcomes remain largely comparable to DN alone, and frequency variations show no consistent advantage. Overall, electroacupuncture emerges as a clinically promising adjunct, though its distinctive therapeutic contribution requires further clarification through longer-term trials [36,37]. These results align with earlier systematic reviews that also found short-term analgesic benefits of electroacupuncture for chronic neck pain, though prior evidence was limited by low methodological quality [109].

Manual and Release-Based Methods

Myofascial release techniques, including ischemic compression, cupping, and instrument-assisted methods (IASTM), may provide short-term analgesia and functional gains [35,40,48,55,56]. However, durability is limited, and outcomes vary with practitioner technique. INIT and neurodynamic approaches show greater consistency against modalities such as spray-and-stretch, exercise, or IASTM, particularly when combined with ischemic compression or post-isometric relaxation [75-78,80].

Physical Agents and Electrotherapies

Across available trials, ESWT consistently reduced pain and improved PPT, ROM, and disability, showing comparable effects to DN but greater efficacy than kinesiotaping or conventional physiotherapy. Given its non-invasive profile and safety, ESWT is a viable option, though its higher cost may limit accessibility and availability [41,45,71,81,82,110].

Based on current evidence, TECAR therapy demonstrates potential analgesic and functional benefits in patients with myofascial pain, including reductions in pain and disability and improvements in cervical mobility. However, randomized trials show inconsistent superiority over placebo, exercise, or therapeutic ultrasound, with gains in both intervention and control groups often suggesting nonspecific effects [84-86]. Given these mixed findings, TECAR may be best considered an adjuvant modality, and larger, high-quality trials are needed to clarify its specific efficacy and long-term clinical relevance.

Ultrasound-based interventions, including phonophoresis and high-power pain threshold ultrasound, have shown significant reductions in pain and improvements in cervical mobility and pressure pain thresholds among patients with upper trapezius myofascial pain [40,67,85,87]. Yet, their therapeutic effects are often comparable to other modalities such as dry needling, iontophoresis, diathermy, or vibration therapy, limiting claims of clear superiority. Thus, ultrasound modalities may serve as supportive adjuncts within multimodal care rather than primary interventions.

Electrical stimulation therapies, including TENS, interferential current, and microcurrent, demonstrate consistent reductions in pain and disability while improving cervical function in myofascial neck pain [88-91]. However, treatment efficacy appears contingent on stimulation frequency and waveform characteristics, with approaches such as acupuncture-like TENS or low-frequency IFC producing superior outcomes in range of motion and symptom relief compared to conventional TENS. Overall, electrical stimulation may serve as an effective adjunct, though heterogeneous protocols and variable responses highlight the need for standardized application and longer-term evaluation.

While laser therapy demonstrates promising analgesic and functional benefits, evidence remains heterogeneous, with some trials indicating only modest or short-term effects. Variability in wavelength, dosage, and treatment protocols complicates comparisons and weakens consensus on optimal clinical application. Collectively, the findings suggest laser therapy may serve as a useful adjunct rather than a definitive intervention, warranting further rigorously controlled investigations [52,65,92-95].

Exercise-Based Strategies

Exercise is the most consistent contributor to sustained improvement in pain, mobility, and function. Although traditionally regarded as a core component of conventional physiotherapy care, most of the protocols tested in recent trials differ in their intensity, specificity, and progression from what is routinely prescribed in everyday practice. These programs emphasize motor relearning by retraining impaired cervical-scapular coordination and the endurance of deep neck and shoulder girdle muscles under low-load conditions, as required for postural support and joint control. Other protocols include targeted exercises such as cervical ROM, isometric chin-in, upper trapezius stretching, scapular retraction, and strengthening drills (e.g., bent-over rows, reverse flies) [53,72,73]. These advanced exercise paradigms consistently yield sustained improvements in pain, mobility, and function, both as stand-alone treatments and when combined with modalities such as DN or myofascial release. Exercise also reduces trapezius trigger point thickness and recurrence risk, underscoring its preventive potential. Thus, while exercise belongs to conventional care, its optimal implementation in MPS management requires a higher level of tailoring and clinical expertise than is often provided in standard practice.

Clinical Implications and Evidence Gaps

The evidence clearly supports a multimodal framework, exercise as the foundation, augmented by DN or ESWT for short- and medium-term gains, and complemented by manual or electrotherapies depending on patient preference, resources, and clinical setting. High-tech modalities such as ESWT, TECAR, and lasers show promise but require stronger cost-effectiveness analyses and longer follow-up trials before broad adoption. Across all modalities, heterogeneity in treatment protocols, session frequency, and outcome measures limits comparability. Few trials extend beyond three months, and head-to-head comparisons remain scarce, leaving uncertainty over optimal sequencing of interventions.

Despite consistent positive findings, an important gap is that most trials were comparative in nature, evaluating one active modality against another rather than including sham or no-treatment controls. Across trials, no serious adverse events were reported; however, minor effects such as transient soreness or bruising were occasionally noted following dry needling, ESWT, and other manual techniques. Adverse event reporting was inconsistent, limiting firm comparison of modality-specific safety profiles. Nonetheless, current evidence supports the clinical usefulness of multiple advanced physiotherapy modalities for cervical MPS. Further trials with standardized comparators and longer follow-up are required to clarify optimal sequencing and integration into care pathways.

Limitations

This review has several limitations. The literature search was restricted to two databases (PubMed and PEDro), which may have led to the omission of relevant studies indexed elsewhere. Grey literature, conference abstracts, and unpublished data were not considered, introducing the possibility of publication bias. The review was also limited to English-language RCTs published between 2019 and 2025, which may narrow its comprehensiveness. In addition, many included trials lacked placebo or sham control groups and relied on direct comparisons between active interventions. Finally, the heterogeneity of study designs, treatment protocols, and outcome measures reduces comparability and limits the generalizability of the findings. Despite these boundaries, the review has notable strengths. To our knowledge, it is among the few to narratively synthesize and compare a wide range of advanced physiotherapy modalities beyond standard care for cervical myofascial pain. By considering interventions collectively, it highlights comparative trends and potential synergies that are not visible when modalities are examined in isolation. Future systematic reviews and meta-analyses are warranted to evaluate these studies more rigorously and to establish evidence-based hierarchies of effectiveness, ultimately facilitating the management of chronic neck pain.

## Conclusions

This review highlights that advanced physiotherapy interventions, particularly DN, ESWT, manual therapies, and specialized exercise approaches, which are often considered beyond standard care, appear beneficial for cervical MPS. DN is the most extensively studied intervention, with many trials reporting positive short- and medium-term outcomes. However, its predominance in the literature reflects both research emphasis and accessibility of the technique and cannot alone be taken as proof of superiority over other modalities that remain less investigated. Exercise contributes to long-term improvements in pain, function, and mobility, while manual therapies and physical modalities are associated with short-term relief. Evidence suggests that multimodal approaches, integrating exercise with manual or instrumental techniques, tend to yield favorable outcomes. For clinicians, structured exercise should be prioritized, with adjunct modalities selected according to patient profile and resource availability. Nevertheless, variability in protocols and outcome measures underscores the need for high-quality RCTs with standardized methodologies to establish clear clinical guidelines.
